# Supplementary material for: Aerial photogrammetry and tag-derived tissue density reveal patterns of lipid-store body condition of humpback whales on their feeding grounds
Source: Proc Biol Sci. 2021 Jan 27;288(1943):20202307. doi: 10.1098/rspb.2020.2307 (PMC7893258; doi:10.1098/rspb.2020.2307)
Supplement: Electronic supplementary materials [file rspb20202307supp1.pdf]

Electronic supplementary materials for the manuscript:

Aerial photogrammetry and tag-derived tissue density reveal patterns of lipid-store body condition of humpback whales on their feeding grounds

Kagari Aoki, Saana Isojunno, Charlotte Bellot, Takashi Iwata, Joanna Kershaw, Yu Akiyama, Lucía M. Martín López, Christian Ramp, Martin Biuw, René Swift, Paul Wensveen, Patrick Pomeroy, Tomoko Narazaki, Ailsa Hall, Katsufumi Sato, Patrick J. O. Miller

Contents

Data collection of time-series behavioural data
Time-series data analyses
Data collection of aerial photogrammetry images
Data process of aerial photogrammetry images
Determination of gender and pregnancy
Data processing and Bayesian estimation for the hydrodynamic model
Model fitting and prediction for underlying tissue body density
The most parsimonious Bayesian model for estimates of tissue body density using hydrodynamic analysis
Repeated measurements of TBD and changes in gliding patterns corresponded with TBD

Table S1. Definition of scores used to select each photograph attribute to measure body condition for analyses.

Table S2. Detailed information of 59 tagged humpback whales used for tissue body density estimation using hydrodynamic glide model and the Bayesian estimates from the best model.

Table S3. Detailed information of 55 humpback whales used for the Length-Standardized Surface Area Index from overhead images obtained using aerial photogrammetry data.

Fig. S1. Changes in gliding patterns corresponded with tissue body density of the same individuals (ID H002)

Data collection of time-series behavioural data

Three types of animal-borne recorders were housed within suction-cup attached tags (see Aoki et al. 2012 for details of suction-cup attached tags): acceleration and speed data-loggers W-3MPD3GT (Little Leonardo Co., Tokyo, Japan), ORI-3MPD3GT (Little Leonardo Co., Tokyo, Japan) and sound and movement recording DTAGs (Woods Hole Oceanographic Institution, MA, USA). The W-3MPD3GT logger recorded depth, water temperature, 3-axis magnetic field strength and speed from a flywheel at 1-s intervals and 3-axis acceleration at 32 Hz. The ORI-3MPD3GT logger recorded depth, water temperature, speed from a flywheel at 1-s intervals and 3-axis acceleration and 3-axis magnetic field strength at 20 Hz. The version-2 DTAGs measured pressure, water temperature, 3-axis acceleration and 3-axis magnetic field strength at 50 Hz, which was later down-sampled to 25 Hz. We modified suction-cup attached tags (Type B, see supplement of Aoki et al. 2012 for details) to fit the recorder types (W-3MPD3GT or ORI-3MPD3GT). Tagging was conducted from a small motorboat (<10 m) using either a 5 m hand-pole or a pneumatic launching system (Aerial Rocket Transmitter System, ARTS; Kleivane 1998), which has a greater effective tagging range up to 12–15 m).

Time-series data analyses

Dive data were analysed using the software IGOR Pro (Wave-Metrics Inc., Lake Oswego, OR, USA) and MATLAB (MathWorks Inc., Natick, MA, USA). All dives (maximum depth: ≥4 m) were divided into descent, bottom and ascent phases based on the pitch angle of tagged animals (Miller et al., 2004). Because accelerometers were not attached exactly parallel to the axes of a whale, we corrected possible off-axis placement on the body, following Johnson and Tyack (2003). Acceleration in the tri-axis (longitudinal, lateral, and dorso-ventral axes) directions was divided into components related to the body orientation of the animal with respect to gravity (gravity-based components) and propulsive activities imposed by fluke thrust (Sato et al. 2007). Lower frequency acceleration (mostly gravity-based) of the longitudinal axis was used to calculate the pitch of a whale (Sato et al. 2003). Higher-frequency acceleration of the dorso-ventral and/or longitudinal axis was used to identify fluke strokes (Sato et al. 2007; for details also see Aoki et al. 2017, Narazaki et al. 2018). According to the power spectral density of each axis (Sato et al. 2007), fluke stroking was determined when oscillation on the dorso-ventral and/or longitudinal axis of the accelerometer exceeded a threshold that was set manually for each deployment. For DTAG data, we objectively separated fluke strokes from glides using a log-survivorship plot of the dorso-ventral and/or longitudinal acceleration. Using the first inflection point in the slope of the log-frequency plot as the threshold.

Speed through the water was measured using an external propeller on the Little Leonardo loggers. The propeller rotation count was converted to actual swimming speed ($m \cdot s^{-1}$) by using a calibration line obtained from a linear regression of rotation rate against swim speed ($m \cdot s^{-1}$). This was calculated from the rate of change in vertical depth divided by the sine of the pitch (Sato et al. 2003) when sine |pitch| was ≥ 0.5 radians. The DTAGs lacked speed sensors; therefore, speed was estimated using the rate of change in depth divided by the sine of pitch when sine |pitch| was ≥ 0.5 radians (Miller et al., 2004).

Data collection of aerial photogrammetry images

We flew an unmanned aerial vehicle (UAV, DJI Phantom 4, DJI Co., Ltd., ShenZhen, China) above individual tagged whales in 2016-2018. The UAV was flown using the DJI GO App as the interface through an android tablet. We used video footage (4096 × 2160 mega-pixel) to capture the entire surfacing sequence of a whale.
The UAV flew at a target elevation of 16–20 m above sea level during flights, while the research vessel followed the targeted individual at a low speed (less than 5 knots) roughly 200 m away. Typically, UAV flights were only made after a suction-cup tag had been attached to the primary target for imaging. Once the UAV was airborne, the targeted animal was followed to obtain aerial images and to capture surrounding animals in the same frame as the tagged whale.

Determination of gender and pregnancy

Collection of biopsy samples from tagged animals enabled both determination of gender and, in the case of females, their reproductive status. Gender was determined through the analysis of skin DNA collected with the blubber biopsy sample (Rosel 2003). Female reproductive status was determined based on progesterone (the primary hormone necessary to maintain pregnancy) concentrations measured in the blubber to classify females as either pregnant or resting (Mansour et al. 2002, Kellar et al. 2006, Pallin et al. 2018), and visual observations of the presence of a calf was used to classify females as lactating. In the Gulf of St Lawrence in Canada, additional information on the age and sex of tagged individuals was available through the long-term photo-ID database of known individuals. Using a combination of archived biopsy samples and the well-established catalogue of photo-identified humpback whales with recorded sightings, identification of pregnancy status has also been validated for the Canadian population of interest here (Kershaw et al. 2020). For detailed hormone extraction, quantification, and quality control procedures to establish pregnancy probabilities, see Kershaw et al. (2020).

Data process of aerial photogrammetry images

Video footage was edited using VLC media player 3.0.2. Individual frames were extracted using Free Video to JPG Converter 5.0.101.201. The best quality frames were selected relying on the following predetermined criteria: (1) posture of the individual, (2) brightness of the image and (3) the animal relative to the surface (Table S1). We extracted a few video frames per individuals during surfacing and allocated scores (1-3, poor to good) of each three criteria. The best photo that had highest averaged score was used to estimate LSSAI. Because of rarely flat calm seas and the behaviour of the whales on feeding ground, it was more challenging than in their breeding ground areas where they spend substantial time logging at the surface (Christian et al., 2016). Therefore, our measurement contained noise, as for any empirical measurement, but was sufficient to see variation in individuals (Fig. 2) and the relationship between LSSAI and tissue body density (Fig. 4).

Frames judged to be of usable quality to measure relative projected body dimensions were measured in detail using a script in R studio 1.1.447, initially provided by Christiansen et al. (2016), and modified for use in this study. We marked the position of the tip of the rostrum and the notch of the flukes, which are considered to represent the total length of the whale (in pixels). The R script then automatically calculated the body axis and created lines perpendicular to the body axis, dividing the length (from the rostrum tip to the fluke notch) into 20 equal sections (Fig. 2, left). We then marked the external boundary of the whale along each line, to create an outline of the whale's body shape, allowing the width of the whale (in pixels) to be measured at each location.

The DJI Phantom 4 has a wide angled lens that potentially generates strong edge effects (distortion). Since we used relative measures of the projected area versus the length, the absolute value of the metrics did not matter, as long as no significant distortion impacted the relative measurements. To check the extent to which lens distortion potentially impacted our dataset, we measured the length and width (at the pectoral fin) of seven whales when the animal was not positioned in the middle of the image (i.e. the animal was at a corner or on the edge of the frame). We compared the original and corrected images using the distortion coefficient of Phantom 4 (Burnett et al. 2018). For absolute measures of length and width, lens distortion effects were <2%. For the width/length ratio, the distortion effect was <1%. Given that these seven animals had the worst possible positions of all of our data, the estimates of lens-distortion errors were sufficiently small to conclude that lens distortion negligibly impacted LSSAI.

Data processing and Bayesian estimation for the hydrodynamic model

We derived the following variables during each 5 s glide from processed time-series tag data and the nearest CTD cast in time and location. Acceleration of the whale body during each 5 s glide was measured using a linear regression line of speed versus time. Pitch (p), seawater density (ρ_{sw}), and speed (v) were averaged during each 5 s sub-glide period. Seawater density (ρ_{sw}) for each sub-glide was calculated from the nearest CTD cast in time and location to each tagged whale. Only stable glides (circular variance of roll and pitch < 0.1) during descent and ascent phases, when the absolute pitch was steeper than 30°, were included in the analysis. Furthermore, we omitted the last 15 s of descent phase and the first 15 s of ascent phases to avoid manoeuvrability gliding which was possibly caused by feeding movements.

The unknown parameters in the hydrodynamic glide model (mainly ρ_{tissue}, $V_{air}m^{-1}$ and C_DAm^{-1}) were estimated by Bayesian Gibbs sampling with the freely available software JAGS within R (coda, R package v0.17-1 2015, http://cran.r.project.org/web/packages/coda/index.html) and R2jags (R package v0.5-7 2012, https://cran.r-project.org/web/packages/R2jags/index.html) using data extracted for each 5 s glide.

For the Bayesian estimation, we set a specific prior distribution for each unknown parameter following a previous study on humpback whales (Narazaki et al. 2018). A non-informative uniform prior was set from 800 to 1200 kg m^{-3} for tissue body density (ρ_{tissue}) and from 5 to 80 ml kg^{-1} for diving gas volume ($V_{air}m^{-1}$), respectively. Based on previous estimations of humpback whales (Narazaki et al. 2018), we specified the prior for C_DAm^{-1} to have a normal distribution with a mean of 11×10^{-6} m^2 kg^{-1} and

standard deviation of 2×10^{-6} m^2 kg^{-1} that was truncated at 1×10^{-6} m^2 kg^{-1} and 29×10^{-6} m^2 kg^{-1} that include the addition of possible effect of induced drag resulting from lift. Observation error measured from the variance of acceleration for each 5 s was incorporated in the model by treating acceleration as a normal variable with a precision parameter (1/variance) (Miller et al. 2016). A small increment (0.001) was added to the standard errors to ensure finite values for the precision parameter.

We explored the variability of unknown tissue density (ρ_{tissue}), combined drag term ($C_D A m^{-1}$) and diving gas volume ($V_{\text{air}} m^{-1}$) by evaluating a total of 12 model structures. We fitted a model in which the value of the unknown parameters ρ_{tissue}, $V_{\text{air}} m^{-1}$ and $C_D A m^{-1}$ remained constant across the tags and dives (global estimates). We also fitted hierarchical models in which the individual-specific estimates of tissue density and/or drag term, and the dive-specific estimates for diving gas volume were considered to be sampled from each global (i.e. individual-average or dive-average) distribution that was estimated for each parameter. See the JAGS script in the appendix of Miller et al. (2016) for the detailed structure of the hierarchical model. All models were sampled in three independent chains, with 48,000 iterations each. The first 24,000 samples were discarded for burn-in, and the remaining posterior samples were downsampled by a factor of 36 to remove any serial correlation in the samples. The best model was selected based on the deviance information criterion (DIC), with a lower value indicating a better model fit relative to model complexity.

Model fitting and prediction for underlying tissue body density

The supplementary R script (AokiEtAl2020_TBD_model.R) is accompanied with the data that is necessary to fit the model (as a csv file, AokiEtAl2020_TBD_data.csv). To run the R script, place the csv file in your working directory and install packages R2jags and lattice. The jags model specification file (TBD_model_v1.txt) is generated as part of the R script and will be saved in your working directory. The script will also generate a pdf to check the convergence of the model (Model_convergence_v1.pdf), as well as posterior estimates (Model_estimates_v1.csv, Model_predictions_v1.tiff).

The most parsimonious Bayesian model for estimates of tissue body density (TBD) using hydrodynamic analysis

A total of 6602 glides were successfully identified at depths ranging from 4 m to 181 m during descent and ascent, with a wide range of swim speeds being documented (range: 0.5–3.9 m s^{-1}). Most tag records contained more than 50 glides (range: 8–605; Table S2).

Bayesian model estimates (means of the posterior distributions) were compared across the various models, which differed in how TBD, diving air volume, and drag coefficient terms were allowed to vary between individuals and dives. The most parsimonious model with the lowest Deviance Information Criterion (DIC) was global (i.e. individual-average) plus individual variation in TBD and drag terms, and global plus dive-by-dive variability in diving lung volume, as found in previous studies using

this method (Miller et al. 2016, Aoki et al. 2017, Narazaki et al. 2018). The model had a DIC value of -6166, decreasing from a DIC of 214417 when the model only contained global values (i.e. fixed values) for all three terms.

Repeated measurements of TBD and changes in gliding patterns corresponded with TBD

Female H002: TBD of this whale was higher (1050.9 kg m^{-3}, very low lipid stores) when resting (not pregnant and lactating) during the early part of the feeding season in 2016, compared to when it was pregnant in the mid feeding season in 2011 (1027.9 kg m^{-3}). When resting, H002 predominantly employed gliding during descent (glide ratio, 77 ± 32% descent, 32 ± 21% ascent, n = 9 dives). When pregnant, however, H002 glided more during ascent (glide ratio, 25 ± 24 % descent, 40 ± 31% ascent, n = 57 dives, Fig. S1). This indicates that the relatively large lipid-stores caused the whale to be positively buoyant. Besides the higher lipid stores during pregnancy, positive buoyancy could also result from a large residual air volume. However, during H002's pregnancy, mean residual air volume was smaller (23.0 ± 10.3 ml kg^{-1}, n = 19 dives) than when it was resting (39.3 ± 16.0 ml kg^{-1}, n = 9 dives). This indicates that large lipid content caused the whale to be positively buoyant.

Female H584: TBD of this whale was relatively low (1028.8 kg m^{-3}) when pregnant during mid feeding season in 2011, indicating a large lipid-store. When lactating during the early part of the feeding season in 2017, H584's TBD was higher (1035.7 kg m^{-3}, i.e. relatively low lipid-store) than during 2011. When lactating, H584 predominantly employed gliding during descent (50 ± 31 % descent, 35 ± 24% ascent, n = 129 dives, Fig. 3), while she glided more during ascent when pregnant (5.5 ± 14% descent, 16 ± 21% ascent, n = 32 dives, Fig. 3). Residual air was also less (29.7 ± 5.9 ml kg^{-1}, n = 8 dives) when pregnant with relatively low TBD compared to when lactating with relatively high TBD (39.2 ± 10.0 ml kg^{-1}, n = 113 dives).

Table S1. Definition of quality scores used to select each photograph attribute used to measure LSSAI.

**Attribute** (criteria)	**Score 1** (poor)	**Score 2** (medium)	**Score 3** (good)
(1) Animal posture (straightness, arch)	The rostrum of the whale is not aligned with the notch of the fluke vertically and horizontally.	The rostrum of the whale is not aligned with the notch of the fluke vertically or horizontally.	The whale is straight both vertically and horizontally.
(2) Brightness	Brightness is high with reflections on the water surface.	Brightness is intermediate and slightly reduces visibility.	Brightness is good, the body shape of the whale is clearly visible.
(3) Depth (animal relative to the surface)	The animal is completely submerged or the animal has just dived, which blurs body shape.	The animal is surfacing between breaths, but there are ripples in the water that partly obscure body shape.	The animal is underwater, but the body shape is clearly visible and there are no ripples, or the animal has half of the back end out, but weak rippling makes the body visible.

Table S2. Detailed information of 59 tagged humpback whales used for tissue body density estimation (ρ_{tissue}) using hydrodynamic glide model and individual-specific estimates from the best model (No.12). These data included 32 whales from the Canadian feeding ground (females: 5 pregnant, 1 lactating, 11 resting, 1 immature; males: 10 mature, 3 immature; 1 unsexed adult) and 27 whales from the Norwegian feeding ground (females: 5 pregnant, 1 pregnant and lactating, 3 resting, 3 immature; males: 5 mature, 1 mature or immature; 4 unsexed adults, 3 unsexed immatures; 2 unknown individuals).

Tag ID	Whale ID	Date (DD/MM/YYYY)	Location	Duration (h)	Tag type	Age class	Sex**	No. of 5 sec glides	ρ_{tissue} (kg m^{-3})	C_DAm^{-1} (x10^{-6} m^2 kg^{-1})
Mn11_H607*	H607	22/07/2011	Canada	3.4	W-3MPD3GT	Adult	M	39	1037.6 ± 3.2	12.2 ± 2.75
Mn11_H686	H686	25/07/2011	Canada	4.5	W-3MPD3GT	Adult	F	70	1036.3 ± 2.5	6.5 ± 4.0
Mn11_H761	H761	25/07/2011	Canada	5.9	W-3MPD3GT	Adult	M	36	1030.6 ± 4.2	22.4 ± 14.6
Mn11_H731	H731	26/07/2011	Canada	2.7	W-3MPD3GT	Adult	F	56	1035.7 ± 2.5	13.4 ± 3.9
Mn11_H698	H698	26/07/2011	Canada	2.0	W-3MPD3GT	Adult	M	58	1032.8 ± 2.7	32.1 ± 14.9
Mn11_H584*	H584	28/07/2011	Canada	3.6	W-3MPD3GT	Adult (Pregnant)	F	46	1028.8 ± 1.3	11.8 ± 2.9
Mn11_H707	H707	19/08/2011	Canada	1.6	W-3MPD3GT	Juvenile	M	98	1043.5 ± 2.7	12.3 ± 2.8
Mn11_H755	H755	28/08/2011	Canada	2.9	W-3MPD3GT	Juvenile	M	177	1032.0 ± 1.0	24.6 ± 1.7
Mn11_H607_Sept*	H607	01/09/2011	Canada	2.1	W-3MPD3GT	Adult	M	29	1032.5 ± 4.6	19.8 ± 24.0
Mn11_H002*	H002	04/09/2011	Canada	5.8	W-3MPD3GT	Adult (Pregnant)	F	184	1027.9 ± 1.2	6.7 ± 6.0
Mn11_H405	H405	18/09/2011	Canada	3.3	W-3MPD3GT	Adult	M	89	1033.7 ± 1.5	12.5 ± 1.7
Mn11_157a	-	06/06/2011	Norway	16.6	Dtag	Adult (Pregnant)	F	77	1039.4 ± 3.0	10.5 ± 11.7
Mn11_158a	-	07/06/2011	Norway	10.2	Dtag	Adult	U	295	1042.1 ± 2.0	11.2 ± 3.6
Mn11_160a	-	09/06/2011	Norway	16.3	Dtag	Juvenile	U	313	1043.4 ± 1.8	12.5 ± 2.7
Mn11_165d	-	14/06/2011	Norway	17.8	Dtag	Juvenile	U	40	1040.5 ± 1.4	17.0 ± 1.6
Mn11_165e	-	14/06/2011	Norway	13.0	Dtag	Adult	M	278	1035.1 ± 1.5	10.8 ± 3.5
Mn11_176b	-	24/06/2011	Norway	4.7	Dtag	Adult	U	50	1039.2 ± 4.1	2.7 ± 5.6
Mn12_158a	-	06/06/2012	Norway	12.8	Dtag	Juvenile	U	605	1039.3 ± 1.2	10.3 ± 1.6
Mn12_164a[s]	-	12/06/2012	Norway	9.3	Dtag	Adult	F	18	1041.6 ± 7.0	8.7 ± 19.3
Mn12_164b[s]	-	12/06/2012	Norway	8.6	Dtag	Juvenile	F	19	1042.5 ± 7.1	6.1 ± 13.6
Mn12_170a[s]	-	12/06/2012	Norway	16.8	Dtag	Juvenile	F	157	1037.3 ± 2.2	5.0 ± 6.4
Mn12_170b[s]	-	18/06/2012	Norway	15.9	Dtag	Adult (Pregnant)	F	153	1037.6 ± 2.0	7.5 ± 3.5
Mn12_171b[s]	-	19/06/2012	Norway	17.3	Dtag	Adult	M	51	1047.6 ± 2.3	0.3 ± 0.8
Mn12_178a[s]	-	26/06/2012	Norway	8.6	Dtag	Adult	F	91	1049.6 ± 0.9	11.9 ± 1.9
Mn12_180a[s]	-	28/06/2012	Norway	15.0	Dtag	Adult (Prengnant,Lactating)	F	85	1047.4 ± 3.9	14.0 ± 7.6
Mn13_Kvaloya7	-	06/12/2013	Norway	3.7	W-3MPD3GT	Unknown	M	30	1031.7 ± 2.2	19.4 ± 7.6
Mn14_121r	-	20/11/2014	Norway	9.2	W-3MPD3GT	Unknown	U	85	1039.8 ± 2.4	11.9 ± 4.2
Mn16_175a	H140	23/06/2016	Canada	11.3	W-3MPD3GT	Adult	F	88	1032.9 ± 1.6	10.1 ± 4.2
Mn16_177a*	H002	25/06/2016	Canada	1.3	W-3MPD3GT	Adult	F	31	1050.9 ± 5.9	37.0 ± 8.5
Mn16_178a	-	29/06/2016	Canada	0.7	W-3MPD3GT	Juvenile	F	10	1043.0 ± 8.4	17.5 ± 12.3
Mn16_248a	H404	04/09/2016	Canada	8.6	ORI-3MPD3GT	Adult	M	349	1041.4 ± 1.9	12.0 ± 5.9
Mn16_250a	H494	06/09/2016	Canada	0.8	ORI-3MPD3GT	Adult	F	28	1036.8 ± 4.7	15.7 ± 4.6
Mn16_258a	H109	14/09/2016	Canada	5.8	ORI-3MPD3GT	Adult	F	49	1028.8 ± 3.2	16.1 ± 6.4
Mn16_Jan19a	-	19/01/2016	Norway	3.2	W-3MPD3GT	Unknown	U	67	1039.8 ± 1.5	20.4 ± 4.1
Mn16_Jan25b	-	25/01/2016	Norway	14.7	W-3MPD3GT	Adult	U	335	1037.8 ± 1.0	18.0 ± 1.5
Mn16_017a	-	17/01/2016	Norway	4.1	Dtag	Adult	M	19	1035.0 ± 5.1	8.5 ± 10.9
Mn16_021a	-	21/01/2016	Norway	5.4	Dtag	Adult	F	29	1034.4 ± 0.9	6.5 ± 1.7
Mn16_023a	-	23/01/2016	Norway	3.4	Dtag	Adult	M	14	1035.8 ± 7.9	10.2 ± 22.3
Mn17_022a	-	22/01/2017	Norway	7.6	W-3MPD3GT	Adult (Pregnant)	F	35	1029.1 ± 1.2	10.8 ± 3.5
Mn17_022LLb	-	22/01/2017	Norway	9	W-3MPD3GT	Adult (Pregnant)	F	117	1030.2 ± 0.9	14.3 ± 3.4
Mn17_026LLa	-	26/01/2017	Norway	15	W-3MPD3GT	Adult (Pregnant)	F	14	1033.9 ± 3.5	4.3 ± 6.7
Mn17_018a	-	18/01/2017	Norway	3.5	Dtag	Adult	M	53	1040.5 ± 0.8	9.8 ± 0.9
Mn17_026a	-	25/01/2017	Norway	5.6	Dtag	Adult	U	84	1035.4 ± 1.2	7.6 ± 2.5
Mn17_158a	-	06/06/2017	Canada	12.1	W-3MPD3GT	Adult	F	83	1030.6 ± 1.8	8.2 ± 4.5
Mn17_165a	H102	12/06/2017	Canada	26.5	W-3MPD3GT	Adult (Pregnant)	F	131	1032.5 ± 2.2	4.0 ± 6.0
Mn17_174a	H456	21/06/2017	Canada	39.8	W-3MPD3GT	Adult (Pregnant)	F	177	1034.0 ± 1.5	20.7 ± 4.3
Mn17_174b	H854	21/06/2017	Canada	7.5	W-3MPD3GT	Adult	M	69	1035.2 ± 2.9	11.7 ± 6.1
Mn17_178a	H748	25/06/2017	Canada	44.6	W-3MPD3GT	Adult (Pregnant)	F	208	1035.0 ± 1.4	20.4 ± 2.1
Mn17_178c	H008	24/06/2017	Canada	57.6	W-3MPD3GT	Adult	F	215	1041.3 ± 2.1	1.6 ± 3.7
Mn17_179a	H840	26/06/2017	Canada	11.2	W-3MPD3GT	Juvenile	M	108	1038.3 ± 2.7	16.1 ± 4.1
Mn17_180a	H007	27/06/2017	Canada	9.4	W-3MPD3GT	Adult	M	133	1037.1 ± 2.2	16.0 ± 3.5
Mn17_180b	H777	29/06/2017	Canada	12.8	W-3MPD3GT	Adult	F	92	1043.6 ± 2.9	20.5 ± 3.9
Mn17_184a	-	03/07/2017	Canada	18.6	W-3MPD3GT	Adult	F	103	1038.6 ± 2.6	17.9 ± 3.9
Mn17_186b	H152	05/07/2017	Canada	11.6	W-3MPD3GT	Adult	M	101	1038.8 ± 3.0	6.1 ± 4.5
Mn17_186c	H151	05/07/2017	Canada	6.6	W-3MPD3GT	Adult	M	134	1037.6 ± 2.0	10.1 ± 3.4
Mn17_186d	-	05/07/2017	Canada	6	W-3MPD3GT	Adult	F	68	1031.8 ± 2.2	20.3 ± 4.6
Mn17_190a*	H584	07/07/2017	Canada	19.1	W-3MPD3GT	Adult (Lactating)	F	277	1035.7 ± 1.7	14.4 ± 2.6
Mn17_191a	H841	08/07/2017	Canada	29.4	W-3MPD3GT	Adult	U	111	1033.0 ± 2.3	11.2 ± 5.8
Mn18_013a	-	13/01/2018	Norway	5.34	W-3MPD3GT	Juvenile	F	41	1042.0 ± 1.7	15.3 ± 2.5

* of Tag ID colum: Measured twice across seasons or years in Canada (ID H002, H584, H607).

s of Tag ID colum: Sound exposure experiments in Norway.

**F, M and U of Sex column: Female, Male, Unknow sex, respectively

Table S3. Detailed information of 55 humpback whales used for the Length-Standardized Surface Area Index (LSSAI) from overhead images obtained using aerial photogrammetry data (7 lactating females, 6 pregnant females, 10 resting females, 3 immature female, 6 mature males, 1 immature male, 14 mature unsexed individuals, 1 immature unsexed individuals, 5 unsexed calves, 2 unknown individuals). Score indicates relative quality of images (1-3, poor to good).

UAV ID	Tag ID	Location	Julian date	Sex	Age Class	Reproductive Status	UAV elevation (m)	Score	LSSAI
BAD	-	Canada	156	Female	Adult	Lactating	20.1	1.8	0.06193
BADc	-	Canada	156	Unknwon	Calf	Calf	20.1	1.8	0.07070
BBR	-	Canada	157	Female	Adult	Pregnant	27.7	2.5	0.07622
FOF	Mn17_158a	Canada	158	Female	Adult	Resting	28.0	2.0	0.08463
BOL	Mn17_165a	Canada	165	Female	Adult	Pregnant	18.9	2.3	0.07817
FOB	-	Canada	166	Male	Adult	Mature	19.2	2.0	0.07063
JUV	-	Canada	166	Male	Juvenile	Immature	30.5	2.8	0.07727
FAT	Mn17_174a	Canada	174	Female	Adult	Pregnant	25.6	2.0	0.07148
WIL	Mn17_174b	Canada	174	Male	Adult	Mature	22.0	2.5	0.07194
IRI	-	Canada	174	Female	Adult	Lactating	15.0	2.3	0.06887
IRIc	-	Canada	174	Unknwon	Calf	Calf	15.0	2.0	0.06975
FRI	Mn17_178a	Canada	178	Female	Adult	Pregnant	25.6	2.5	0.07907
SOU	-	Canada	178	Female	Adult	Lactating	25.6	2.5	0.07275
SOUc	-	Canada	178	Unknwon	Calf	Calf	26.8	2.8	0.07691
PAL	-	Canada	178	Female	Adult	Resting	27.1	2.3	0.07105
TIN	-	Canada	178	Female	Adult	Lactating	20.4	2.8	0.06575
TINc	-	Canada	178	Unknwon	Calf	Calf	27.1	1.8	0.08114
PSE	Mn17_178c	Canada	178	Female	Adult	Resting	18.2	1.8	0.06810
SIA	Mn17_180a	Canada	180	Male	Adult	Mature	18.9	2.3	0.06968
RAL	Mn17_180b	Canada	180	Female	Adult	Resting	15.2	1.8	0.07399
EYE	Mn17_184a	Canada	184	Female	Adult	Resting	11.0	2.8	0.06846
SPL	-	Canada	186	Female	Adult	Pregnant	16.2	2.8	0.07233
STL	Mn17_186b	Canada	186	Male	Adult	Mature	22.3	2.0	0.06706
SPI	Mn17_186c	Canada	186	Male	Adult	Mature	21.6	2.3	0.07619
FOS	Mn17_186d	Canada	186	Female	Adult	Resting	21.3	2.3	0.07021
MAN	Mn17_190a	Canada	190	Female	Adult	Lactating	22.3	2.8	0.06477
MANc	-	Canada	190	Unknwon	Calf	Calf	22.3	2.0	0.07812
HW1	Mn17_022a	Norway	387	Female	Adult	Resting	16.0	2.0	0.08181
HW2	-	Norway	387	Unknwon	Adult	Mature	16.0	2.3	0.06790
HW3	-	Norway	388	Unknwon	Adult	Mature	16.0	2.3	0.08344
HW5	-	Norway	389	Unknwon	Adult	Mature	14.0	2.8	0.07248
HW6	-	Norway	389	Male	Adult	Mature	14.0	2.8	0.07852
HW7	Mn17_026LLa	Norway	391	Female	Adult	Pregnant	14.0	2.5	0.08713
HW8	Mn17_026a	Norway	391	Unknwon	Adult	Mature	13.0	2.3	0.07129
HW9	-	Norway	391	Unknwon	Adult	Mature	14.0	2.5	0.07994
HW10	-	Norway	391	Unknwon	Adult	Mature	13.0	2.0	0.08238
DAR	Mn16_175a	Canada	175	Female	Adult	Resting	-	1.8	0.07180
SOL	-	Canada	176	Female	Juvenile	Immature	-	2.5	0.06842
HAN	Mn16_178a	Canada	178	Female	Juvenile	Immature	-	2.3	0.06629
FOH	-	Canada	178	Unknwon	Unknwon	Unknwon	-	2.0	0.07089
FOH2	-	Canada	178	Unknwon	Unknwon	Unknwon	-	2.3	0.08204
BOO	Mn16_250a	Canada	250	Female	Adult	Resting	19.0	3.0	0.07924
HEL	-	Canada	250	Unknwon	Adult	Mature	18.9	2.5	0.08137
TRA	Mn16_258a	Canada	258	Female	Adult	Resting	19.0	2.3	0.09396
FOT	-	Canada	258	Unknwon	Adult	Mature	23.0	1.5	0.07007
QUI	-	Canada	265	Unknwon	Adult	Mature	17.0	2.5	0.07178
MAR	-	Norway	376	Unknwon	Adult	Mature	16.0	2.5	0.07447
BIG	-	Norway	376	Unknwon	Adult	Mature	16.0	2.5	0.07732
DOTm	-	Norway	378	Female	Adult	Lactating	16.0	2.5	0.06862
DOT	-	Norway	378	Unknwon	Juvenile	Immature	16.0	1.5	0.07795
ROL	Mn18_013a	Norway	378	Female	Juvenile	Immature	19.0	2.8	0.08406
ROLm	-	Norway	378	Female	Adult	Lactating	16.0	2.3	0.07225
SUZ	-	Norway	378	Unknwon	Adult	Mature	16.0	2.0	0.07589
LAU	-	Norway	378	Unknwon	Adult	Mature	16.0	2.3	0.07152
FRA	-	Norway	378	Unknwon	Adult	Mature	16.0	2.3	0.07008

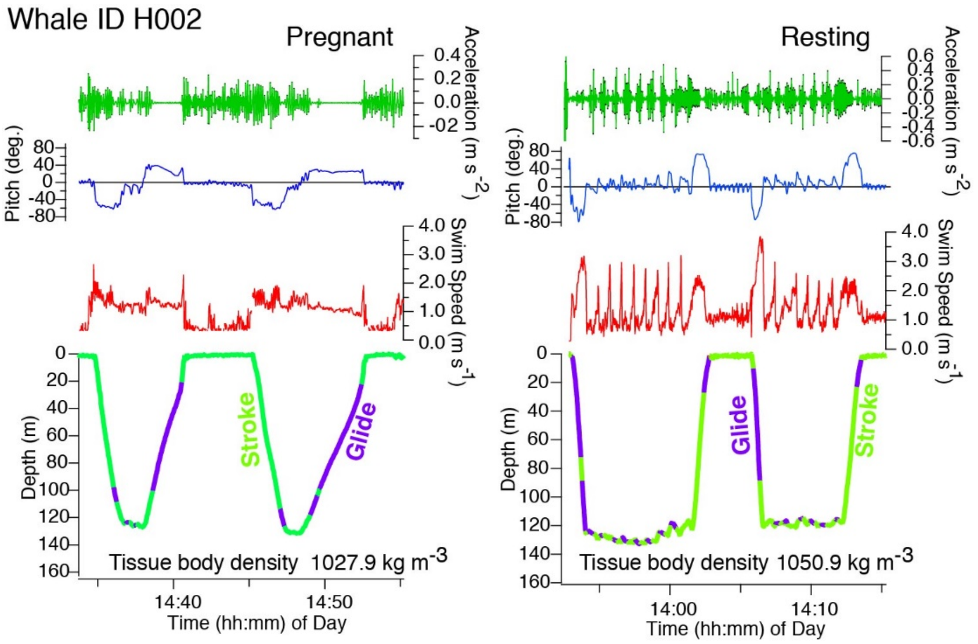

Fig. S1. Changes in gliding patterns corresponded with tissue body density of the same individuals (ID H002, see Fig.3 for ID H584). The reproductive status is shown. Neither pregnant nor lactating is represented as resting. High values of dorso-ventral accelerations indicate periods of fluke-strokes (dive depth in green) while low values indicate gliding periods (dive depth in purple).

References

Aoki K, Amano M, Mori K, Kourogi A, Kubodera T, Miyazaki N. 2012 Active hunting by deep-diving sperm whales: 3D dive profiles and maneuvers during bursts of speed. Mar. Ecol. Prog. Ser. **444**, 289-301 (doi:10.3354/meps09371)

Aoki K, Sato K, Isojunno S, Narazaki T, Miller PJO. 2017 High diving metabolic rate indicated by high-speed transit to depth in negatively buoyant long-finned pilot whales. J. Exp. Biol. **220,** 3802-3811 (doi:10.1242/jeb.158287)

Burnett JD*,* Lemos L*,* Barlow DR*,* Wing MG*,* Chandler TE, Torres LG. 2018 Estimating morphometric attributes of baleen whales with photogrammetry from small UAS: A case study with blue and gray whales*.* Mar. Mam. Sci. **35***,* 108–139.

Christiansen F, Dujon AM, Sprogis KR, Arnould JPY, Bejder L. 2016 Non-invasive unmanned aerial vehicle provides estimates of the energetic cost of reproduction in humpback whales. Ecosphere **7**, e01468 (doi:10.1002/ecs2.1468)

Johnson MP, Tyack PL. 2003 A digital acoustic recording tag for measuring the response of wild marine mammals to sound. IEEE J. Ocean. Eng. **28**, 3-12 (doi:10.1109/JOE.2002.808212)

Kellar NM, Trego ML, Marks CI, Dizon AE. 2006 Determining pregnancy from blubber in three species of delphinids. Mar. Mamm. Sci. **22**, 1-16.

Kershaw JL, Ramp CA, Sears R, Plourde S, Brosset B, Miller PJO, Hall A. 2020 Declining reproductive success in the Gulf of St. Lawrence's humpback whales (*Megaptera novaeangliae*) reflects ecosystem shifts on their feeding grounds. Global Change Biology. (doi.org/10.1111/gcb.15466)

Kleivane L. 1998. A New Pneumatic Launching Device ARTS (Aerial Rocket Transmitter System) Especially Developed and Designed to Improve Tagging and Instrumentation of Baleen Whales. Bodø, Norway: Restech A/S

Mansour AH, McKay D, Lien J, Orr JC, Banoub JH, Oien N, Stenson G. 2002 Determination of pregnancy status from blubber samples in minke whales (*Balaenoptera acutorostrata*). Mar. Mamm. Sci. **18**, 112-120 (doi:10.1111/j.1748-7692.2002.tb01022.x)

Miller PJO, Johnson MP, Tyack PL, Terray EA. 2004. Fluking patterns, passive drag, and buoyancy of diving sperm whales *Physeter macrocephalus*. J. Exp. Biol. **207**, 1953-1967.

Miller PJO, Narazaki T, Isojunno S, Aoki K, Smout S, Sato K. 2016 Body density and diving gas volume of the northern bottlenose whale (*Hyperoodon ampullatus*). J. Exp. Biol. **219**, 2458-2468 (doi:10.1242/jeb.137349)

Narazaki T, Isojunno S, Nowacek DP, Swift R, Friedlaender AS, Ramp C, Smout S, Aoki K, Volker BD, Sato K, Miller PJO. 2018 Body density of humpback whales (*Megaptera novaeangliae*) in feeding aggregations estimated from hydrodynamic gliding performance. PLoS ONE **13**, e0200287 (doi:10.1371/journal.pone.0200287)

Pallin L, Robbins J, Kellar N, Bérubé M, Friedlaender A. 2018 Validation of a blubber-based endocrine pregnancy test for humpback whales. Conserv. Physiol. **6**, coy031 (doi:10.1093/conphys/coy031)

Rosel PE. 2003 PCR-based sex determination in Odontocete cetaceans. Conserv. Genet. **4**, 647-649.

Sato K, Mitani Y, Cameron MF, Siniff DB, Naito Y. 2003 Factors affecting stroking patterns and body angle in diving Weddell seals under natural conditions. J. Exp. Biol. **206**, 1461-1470 (doi:10.1242/jeb.00265)

Sato, K, Watanuki, Y., Takahashi, A., Miller, PJO, Tanaka, H, Kawabe, R, Ponganis, PJ, Handrich, Y, Akamatsu, T, Watanabe, Y. et al. 2007 Stroke frequency, but not swimming speed, is related to body size in free-ranging seabirds, pinnipeds and cetaceans. Proc. R. Soc. B Biol. Sci. **274**, 471-477.
